# Biocontrol and Action Mechanism of *Bacillus amyloliquefaciens* and *Bacillus subtilis* in Soybean Phytophthora Blight

**DOI:** 10.3390/ijms20122908

**Published:** 2019-06-14

**Authors:** Dong Liu, Kunyuan Li, Jiulong Hu, Weiyan Wang, Xiao Liu, Zhimou Gao

**Affiliations:** 1 College of plant protection, Anhui Agricultural University, 130 West of Changjiang Road, Hefei 230036, Anhui, China; liudong1595693@163.com (D.L.); likunyuan9398@163.com (K.L.); jiulonghu2020@163.com (J.H.); wangwy1614@163.com (W.W.); liuxiaolpl@163.com (X.L.); 2 School of life sciences, Anhui Agricultural University, 130 West of Changjiang Road, Hefei 230036, Anhui, China; 3 Department of Horticulture and Landscape, Anqing Vocational and Technical College, 99 North of Tianzhushan Road, Anqing 246003, Anhui, China

**Keywords:** *Phytophthora sojae*, *Bacillus amyloliquefaciens*, *Bacillus subtilis*, bacterial-fungal interactions, transcriptome, induced resistance

## Abstract

With the improper application of fungicides, *Phytophthora sojae* begins to develop resistance to fungicides, and biological control is one of the potential ways to control it. We screened two strains of *Bacillus*; *Bacillus amyloliquefaciens* JDF3 and *Bacillus subtilis* RSS-1, which had an efficient inhibitory effect on *P. sojae.* They could inhibit mycelial growth, the germination of the cysts, and the swimming of the motile zoospores. To elucidate the response of *P. sojae* under the stress of *B. amyloliquefaciens* and *B. subtilis*, and the molecular mechanism of biological control, comparative transcriptome analysis was applied. Transcriptome analysis revealed that the expression gene of *P. sojae* showed significant changes, and a total of 1616 differentially expressed genes (DEGs) were detected. They participated in two major types of regulation, namely “specificity” regulation and “common” regulation. They might inhibit the growth of *P. sojae* mainly by inhibiting the activity of ribosome. A pot experiment indicated that *B. amyloliquefaciens* and *B. subtilis* enhanced the resistance of soybean to *P. sojae*, and their control effects of them were 70.7% and 65.5%, respectively. In addition, *B. amyloliquefaciens* fermentation broth could induce an active oxygen burst, NO production, callose deposition, and lignification. *B. subtilis* could also stimulate the systemic to develop the resistance of soybean by lignification, and phytoalexin.

## 1. Introduction

Soybean Phytophthora root and stem rot, caused by *Phytophthora sojae* has been reported worldwide, for instance, in Asia, Africa, Europe, North America, South America, and so on [1]. *P. sojae* is a destructive soil-borne oomycete pathogen, which is not easy to control and it, therefore, results in great soybean (*Glycine max*) yield reductions. Therefore, the prevention and control of *P. sojae* has been widely studied by researchers. At present, the prevention and control of *P. sojae* is mainly achieved by rationally planting soybean-resistant varieties, spraying effective fungicides, and improving field management measures [2]. However, with the improper application of fungicides, *P. sojae* begins to develop resistance to fungicides, such as metalaxyl, fluorophenylalanine, mefenoxam, zoxamide, phenylamide, and oxathiapiprolin [3,4,5,6,7], and some of the researchers have begun to study their resistance mechanisms. The results showed that the resistance of *Phytophthora* to metalaxyl and mefenoxam was related to RNA polymerase I or its subunit RPA190 [8], and the β-tubulin and oxysterol binding protein from *P. sojae* is related to the resistance of Zoxamide and oxathiapiprolin [4,7]. Moreover, the resistance of *P. sojae* to pharmacy has led to a decrease in the control effect of soybean root rot, which poses a significant potential risk to soybean cultivation. Biological control is one of the effective ways to address this risk [2,9,10]. Wagner et al. [11] found that *Pseudomonas* can colonize well in soybean roots and inhibit *P. sojae*. Moreover, *Pseudomonas* has a good application prospect in controlling soybean Phytophthora blight. According to Godfrey et al. [12] *P. aureofaciens* can also be used as a potential biological control option for *P. sojae.* Fermentation filtrates of *Pseudomonas* sp. BS1 inhibited the zoosporangium formation and zoospore germination of *P. sojae*, and the inhibition rate of stock solutions reaches 95.9% [13]. Sowanpreecha and Rerngsamran [14] found that the antibacterial protein from *P. aeruginosa* RS1 can effectively inhibit *P. palmivora*, and the molecular weight of the antibacterial protein is about 20 kDa to 54 kDa. According to LC/MS, these proteins may be catalase, chitin-binding protein, and protease. Additionally, some proteins from *Pseudomonas* can stimulate the defense reaction of soybean against *P. sojae*, and the over-expression harpin-encoding gene hrpZm from *P. syringae* in soybean enhances tolerance to Phytophthora root and stem rot [15]. Soybean endophytic bacteria, such as *Enterobacter*, *Acinetobacter*, *Pseudomonas*, *Ochrobactrum*, and *Bacillus*, associated with the nodules of soybean, can promote the growth of soybeans and inhibit the pathogenicity of *P. sojae* [16]. Arfaoui et al. [17] found that *Paenibacillus* sp. and *Streptomyces* sp. had a good control effect on *P. sojae* race 4 in vitro. Research shows that the borrelidin from *Streptomyces* sp. neau-D50 plays an important role in the inhibition of *P. sojae*, and the antifungal activity of borrelidin was mediated by the inhibition of threonyl-tRNA synthetase (ThrRS) via the formation of the ThrRS-borrelidin complex [18].

Some *Bacillus*, such as *B. subtilis* and *B. pumilus*, also have good control effects against *P. sojae* [2,12]. *B. altitudinis* JSCX-1 can inhibit *P. sojae* by inhibiting the mycelial growth and zoospore germination. Biocontrol efficiency of *B. altitudinis* JSCX-1 against *P. sojae* was 49.28%, and it reduces the Pathogenicity of *P. sojae* via increasing the ROS production and callose deposition on soybean, and up-regulating the expression of genes in the salicylate-responsive pathway, such as GmPR1a [2]. It has been reported that *Bacillus* can produce some of the secondary metabolites, lipopeptides, and antibacterial proteins, such as aminopeptidase and chitinase, which are the main antibacterial components [9,19]. When *B. amyloidosis* is induced by methyl salicylate secreted by plants, it produces antibiotic *Bacillus* peptide and weathered mycin to help plants to resist *Fusarium oxysporum* [13]. It is important to reveal the inhibitory mechanism of *Bacillus* on *P. sojae*, and this will help us to develop *Bacillus* into bio-fertilizers and bio-pesticide. Transcriptomics has become one of the most widely used high-throughput sequencing technologies due to its low-cost and simple processing compared with proteomics and metabonomics [20]. Transcriptome sequencing technology has been extensively used in the interaction between oomycetes and plant hosts, especially between *P. sojae* and soybean. This technology has proven to be a useful tool for identifying oomycetes and genes associated with infection [21,22,23,24]. In 2012, the oomycete transcriptomics database was established by Tripathy et al. [21], and it includes transcript sequences from *P. sojae* and its host soybean (*P. sojae* mycelia, healthy soybean, and *P. sojae*-infected soybean) as well as Illumina transcript sequences from five *Hyaloperonospora arabidopsidis* libraries. In addition, it also has a complete set of Sanger EST sequences from *P. sojae*, *P. infestans*, and *H. arabidopsidis*, grown under various conditions, and these resources have promoted the rapid development of the research on the pathogenic mechanism of *Phytophthora*. Ye et al. [22] used transcriptomics to exploit the candidate virulence gene, and some effector genes (RxLR, CRN, and NLP) were found to play an important role in the interaction between *P. sojae* and soybean. Differentially expressed genes are analyzed with transcriptomics when the zoospore of *P. sojae* is released at a cold temperature, and some genes encoding phosphatidylinositol kinase and Ca^2+^/calmodulin-dependent protein kinase are involved in the zoospore release [23]. Chen et al. [24] applied RNA-Seq technology to reveal infection-related gene expression changes in *P. capsici*, and a large number of genes associated with pathogenicity were identified, including 98 predicted effector genes (RXLR and CRN effectors). The transcriptional dynamics of *P. infestans* during sequential stages of hemibiotrophic infection of tomato was carried out, and transcriptome data indicated that enzyme inhibitors, proteases, and glycosyl hydrolases are associated with pathogenicity, including the induction of putative cytoplasmic and apoplastic effectors [25].

Transcriptome analysis was also occasionally applied to the study of biological control mechanisms [26,27]. Our research uses transcriptomics to elucidate the response of *P. sojae* under the stress of *B. amyloliquefaciens* and *B. subtilis*, and this will contribute to the biological control molecular mechanism of *P. sojae* and lead to an in-depth understanding of the interactions between rhizosphere bacterial and soil-borne oomycete pathogens.

## 2. Results and Discussion

### 2.1. Inhibition of P. sojae by B. amyloliquefaciens and B. subtilis

**Figure 1:**
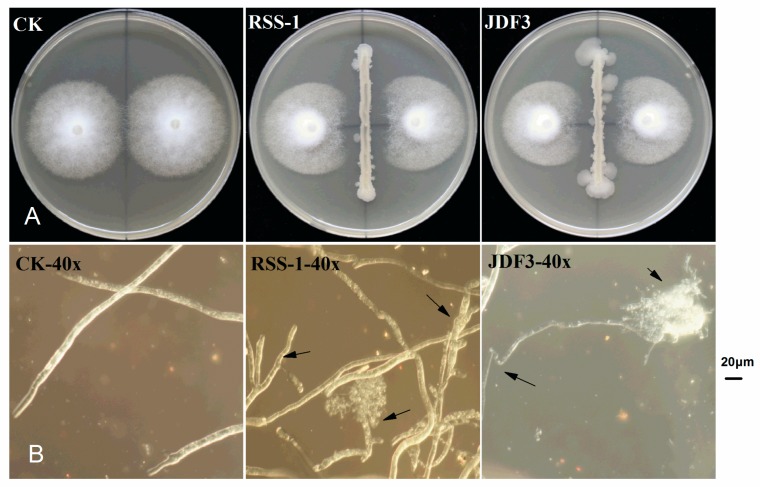
Dual culture in vitro bacterial-fungal assays and morphological changes of mycelium under biocontrol stress. Mycelium morphology was photographed at a 40-fold microscope, and the arrows indicate abnormal hyphae.

**Figure 2:**
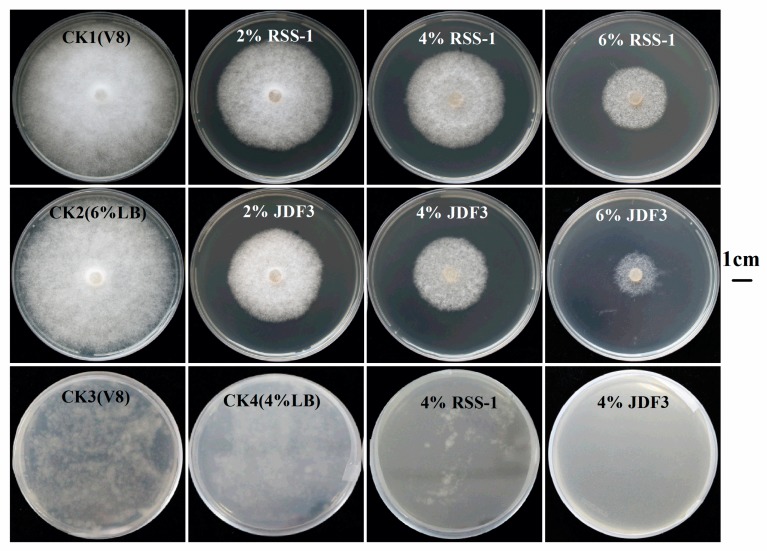
*Bacillus* fermentation broth inhibited the growth and germination of cyst of *P. sojae.* The plates, which contained 2, 4, and 6% *Bacillus* fermentation broth, were used. The CK1 (10% V8), CK3 (10% V8), CK2 (10% V8 + 6% LB), and CK4(V8 + 4%LB) was used as negative and positive control. The third line was the results of *Bacillus* fermentation broth inhibiting the germination of cysts.

**Figure 3:**
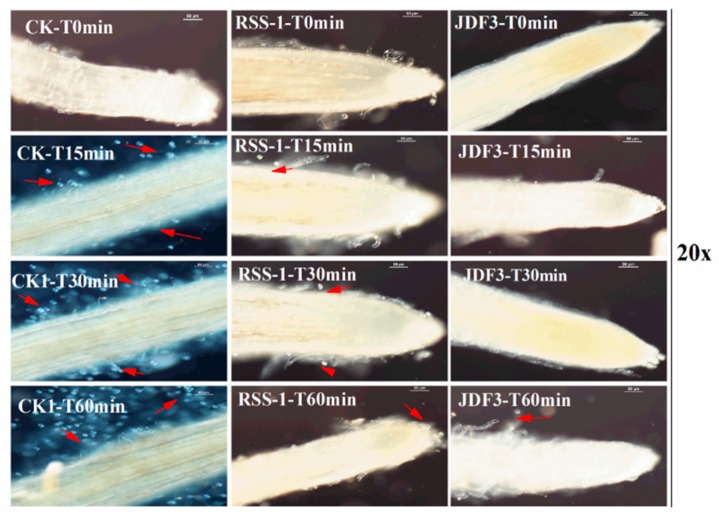
*Bacillus* fermentation broths inhibit the swimming of zoospores. The first column was the results of the adsorption of zoospores on the soybean root after they were placed in the suspension of untreated zoospores for 60 min. The second and third columns were the results of the adsorption of zoospores on the soybean root after they were placed in the suspension of zoospores, which were added into *Bacillus* fermentation broths at 200 μL/20 mL for 10 s. The results were recorded with a 20-fold microscope.

*P. sojae* was cultured for five days with *B. subtilis* RSS-1 and *B. amyloliquefaciens* JDF3. Compared with the control, the growth of *P. sojae* was significantly inhibited, and the inhibition rates were 71.4% and 67.1%, respectively (Figure 1A). Under the microscope, it can be observed that, compared with the normal hyphae, the mycelium of *P. sojae* hyphae showed excessive branching, swelling, and deformity under the stress of two strains of *Bacillus* (Figure 1B). *A. calcoaceticus* had the strong inhibitory activity (71.14%) against the *P. sojae*, which caused fracture, lysis, and the formation of a protoplast ball at the end of hyphae [16]. Zhang et al. [28] reported that melatonin resulted in a branched hyphae, blunt hyphal tip, and a decrease of lipid droplets in *P. infestans.* Benzothiazole is an antimicrobial secondary metabolite volatilized by microbes, and may act as potential leading compound for the development of new oomycete fungicides [29]. Abnormal swellings and branches were developed along the hyphae after exposure to benzothiazole, and the polarized distribution of newly synthesized walls was also disrupted. The abnormal changes in hyphae was due to the disorganized cytoskeleton, and mycelium-related genes were involved in regulation. The fermentation broth of *B. amyloliquefaciens* JDF3 and *B. subtilis* RSS-1 have a good inhibitory effect on *P. sojae* (Figure 2), and the EC50 values for *P. sojae* are 4.06% and 2.79%, respectively. The regression equation of fermentation liquid RSS-1, and JDF3 were Y1 = 1.1059X1 + 4.4103 (R^2^ = 0.9602) and Y2 = 1.2539X2 + 4.5508 (R^2^ = 0.9933), respectively. In addition, their fermentation broth can also inhibit the germination of cysts (Figure 2) and the swimming of motile zoospores (Figure 3), indicating that these two strains of *Bacillus* have a better potential for controlling *P. sojae*.

### 2.2. Transcriptome Sequencing Quality Analysis

In order to study the molecular mechanism of two strains of *Bacillus* inhibiting *P. sojae*, a transcriptome analysis in response to biotic stress was carried out. *P. sojae* (CK), *B. subtilis* vs. *P. sojae* (BST), and *B. amyloliquefaciens* vs. *P. sojae* (BAT), in triplicate, was created, with a sequencing data volume of 6G, and raw reads (150bp, single-end) were obtained. The transcriptome statistics of the nine processed samples are shown in Table 1, and the raw read is in the range of 44,290,410~72,226,262. After the sequencing, the results were evaluated, the low-quality reads were filtered out, and the number of Clean Reads obtained was in the range of 43,117,708~68,948,564. The average number of Clean Reads per sample was 49,584,800. The sequenced read was compared with the *P. sojae* v3.0, and the mapped reads were in the range of 10,625,344~342,99,648. The GC content varied from 48.25–57.73%. In addition, the Q30 value was greater than 83%, indicating that RNA-Seq sequencing is of a good quality and can be used for the analysis of bioinformatics.

### 2.3. Analysis of Principal Components

**Figure 4:**
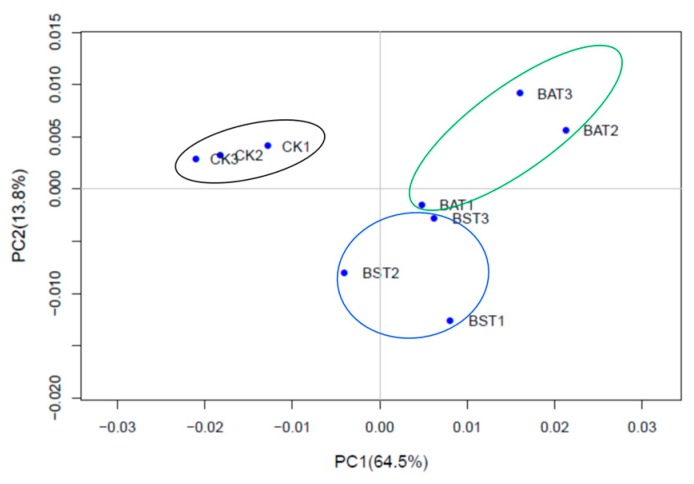
Principal component analysis of the *P. sojae* samples.* The black circle was the control 1 (CK1), control 2 (CK2), control 3 (CK3). The blue circle was *P. sojae* samples treated with *B. subtilis* RSS-1, and BST1, BST2, and BST3 are three biological repeats. The green circle was *P. sojae* samples treated with *B. amyloliquefaciens*, and BAT1, BAT2, and BAT3 are three biological repeats.

To determine the replication of the transcriptome samples, we performed principal component analysis (PCA), and the results are shown in Figure 4. The PCA results revealed that the transcriptome samples were clearly divided into three groups: the control group (CK1, CK2, CK3), *B. amyloliquefaciens* treatment group (BAT1, BAT2, BAT3), and *B. subtilis* treatment group (BST1, BST2, BST3). Three replicate samples within each group were brought together to form an independent population. According to the numerical values of the sample gene expression in the first principal component (PC1) and the second principal component (PC2), a two-dimensional coordinates map of the principal component is drawn. PC1 (64.5%) and PC2 (13.8%) revealed a change in gene expression of 9 samples of 78.3% and showed good agreement between sample biological replicates [22,24].

### 2.4. Effect of B. amyloliquefaciens and B. subtilis on the Gene Expression of P. sojae and KEGG Enrichment Analysis

**Figure 5:**
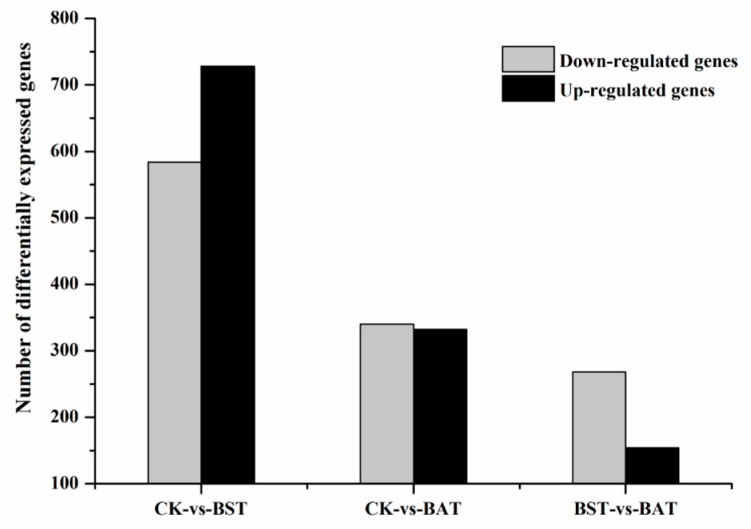
Number of differentially expressed genes of *P. sojae* challenged with *B. amyloliquefaciens* and *B. subtilis* at 25 °C for five days.

**Figure 6:**
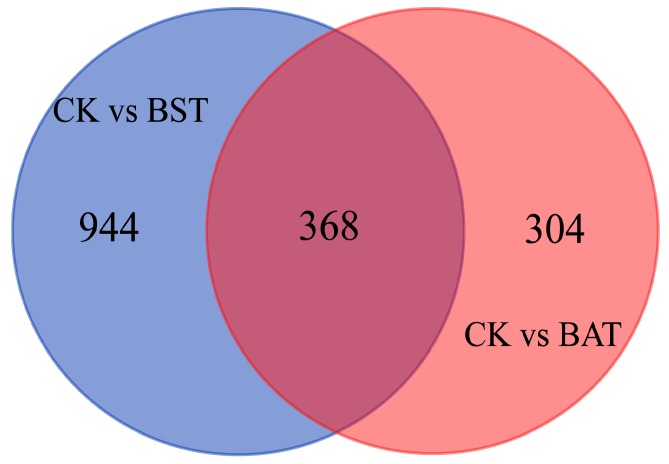
Venn diagram illustrating relationship between the differentially expressed genes of *P. sojae* challenged with *B. amyloliquefaciens* and *B. subtilis* at 25 °C for five days. Intersection is a “common regulation”, and the others are “specific regulation”.

Under the inhibition of two strains of *Bacillus*, the expression of *P. sojae* gene showed significant changes. Under the stress of *B. subtilis* RSS-1, 728 differentially expressed genes (DEGs) were up-regulated, while 584 DEGs were down-regulated. The inhibition of the *B. amyloliquefaciens* JDF3 strain also caused a significant differential expression of the gene, with 332 and 340 genes up-regulated and down-regulated, respectively. Comparing the two strains of *Bacillus* that were treated, it was found that the expression of the *P. sojae* genes was different, and the total number of DEGs was 422, with 154 up-regulated and 268 down-regulated (Figure 5). To learn more about the DEGs between BST and BAT, we showed the differential expression of transcripts in a Venn diagram (Figure 6). In the stressed *P. sojae*, there were 368 transcripts in the intersection, which were generally affected by the biocontrol stress of *B. subtilis* and *B. amyloliquefaciens*, and there were 944 and 304 that were transcript-specific, respectively. Thus, we divided the differentially expressed transcripts into two major types of regulation, namely, “specificity” regulation and “common regulation”.

The commonly regulated genes were enriched in ribosome (ko03010), starch and sucrose metabolism (ko00500), tyrosine metabolism (ko00350), tryptophan metabolism (ko00380), pyruvate metabolism (ko00620), amino sugar and nucleotide sugar metabolism (ko00520), etc. (Figures S1 and S2). The genes enriched in the “ribosome (ko03010)” pathway were all down-regulated. In the BST group, 26 genes were down-regulated, accounting for 26% of the pathway. They involved small ribosomal subunits (S8e, S10e, S20e, S26e, S27e, S27Ae, and S28e, etc.) and large subunits (L9e, L26e, L13Ae, L34Ae, L10Ae, and L36Ae and LP1, LP2e, etc.). In the BAT group, 33 genes were down-regulated, accounting for 33% of the pathway, involving small ribosomal subunits (S4e, S10e, S19e, S20e, S24e, S26e, S27e, S27Ae, and S28e, etc.) and large subunits (L9e, L13Ae, L14e, L34Ae, L35Ae, and L37Ae, etc.). The down-regulated expression of the ribosomal size subunit gene may be caused by the inhibition of the secondary metabolites of *Bacillus*, and the abnormal synthesis of ribosomal proteins may cause the cell proliferation process to be blocked [30,31,32,33]. Moreover, the protein transport pathway (ko03060) was also affected, and PHYSODRAFT_511553 may be involved in the process of protein folding, sorting and degradation.

Sphingolipid acts as a component of the cell membrane, and in the sphingolipid metabolism (ko00600) pathway, the down-regulation of genes affects many functions of the cell membrane, such as membrane transport [34]. In the starch and sucrose metabolism pathway (ko00500) in the above two treatments, the down-regulation DEGs was 10 (in the BST group, Table S2) and 11 (BAT group, Table S3), which account for approximately 6% of the total number of genes in the pathway. This pathway is closely related to the utilization of carbohydrates, energy production and the growth of *P. sojae*, resulting in the inhibition of the growth of *P. sojae* [35,36]. In addition, in the BST group in the “specific regulation” pathway, five down-regulated expression genes were enriched in the fatty acid elongation (ko00062) pathway, while five down-regulated genes were enriched in the fatty acid metabolism (ko01212) pathway, affecting the lipid metabolism pathway. These genes, which were involved in ko04130 (Figure S1, soluble *N*-ethylmaleimide-sensitive factor attachment protein receptor, SNARE), might play an important role in asexual development, sexual reproduction, and pathogenesis like the gene of PsYKT6 in this family [37].

In the BAT group, the other glycan degradation (ko00511) and regulation of autophagy (ko04140) pathways may affect the cell wall production and autophagy regulation of *P. sojae* [38,39]. The down-regulated expression of the regulation of the autophagy pathway may affect the growth and development, sporulation and pathogenicity of *P. sojae* [39], and similar phenomena are observed [40]. Autophagy is ubiquitous in eukaryotic cells and plays an important role in material turnover, which encapsulates damaged organelles or long-lived proteins to form autophagic vesicles [41,42]. After being fused with lysosomes or vacuoles, the substance is degraded and recycled [42]. Two genes (Table S2), which are involved in the pathways of MAPK (ko04011), possibly participated in hyphal growth, zoosporogenesis, cell wall integrity, and pathogenicity like the gene of *PsSAK1* and *PsMPK7* in this family [43,44].

### 2.5. GO Enrichment Analysis

**Figure 7:**
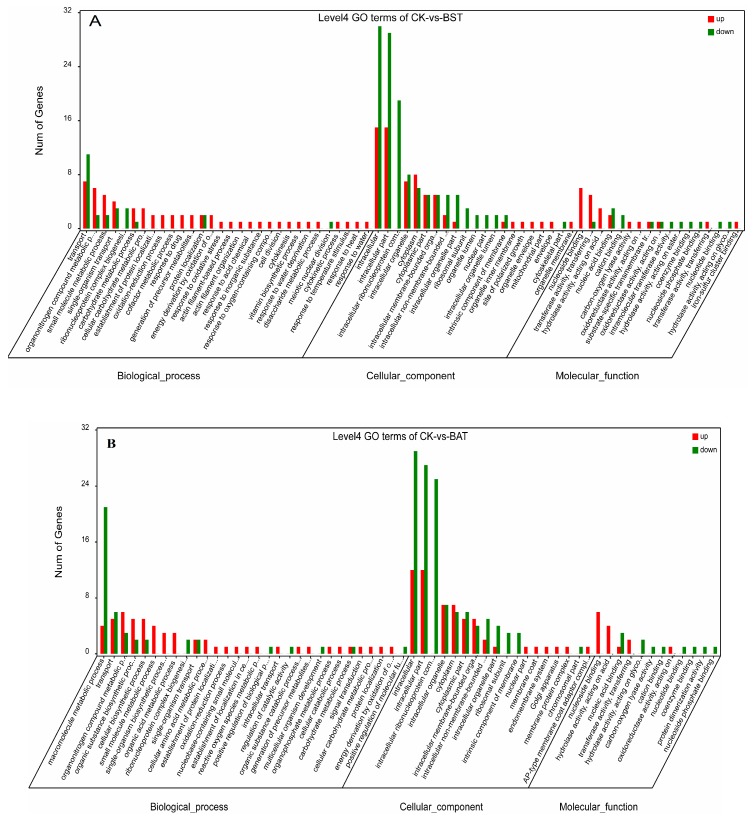
Gene Ontology (GO) enrichment analysis for differentially expressed gene (DEG).

In order to identify the response of *P. sojae* to these two strains of *Bacillus*, we performed a GO functional enrichment analysis on DEGs. The GO enrichment analysis describes the classification of gene functions and the relationships between these genes. The GO functions of DEGs were divided into three groups: The biological process, the cellular component, and the molecular function [26,27]. In the BST samples, 217 up-regulated and 242 down-regulated differentially expressed genes were annotated, respectively. Therefore, a total of 459 DEGs were annotated (Table S4). Among them, 234 DEGs (130 up, 104 down) belong to the biological process (BP) category, 189 DEGs (65 up, 124 down) belong to the cellular component (CC) category, and 36 DEGs (22 up, 14 down) belong to the molecule functional (MF) category (Figure 7A and Table S4).

In the BP, most GO terms were related to transportation, the organonitrogen compound metabolic process, ribonucleoprotein complex biogenesis, and the response to various stresses (e.g., the response to oxidative stress, oxygen-containing compound, temperature stimulus, inorganic substances, heat, water deprivation, etc.). For example, a higher frequency of biological process subcategories is a single-organism cellular process, the regulation of biological process, a cellular response to the stimulus, a macromolecule metabolic process, a heterocycle metabolic process, a small molecule metabolic process, and an emergency response to various external stresses.

For the “cell component” GO category, the higher-frequency subcellular subclasses were the intracellular, intracellular part, intracellular ribonucleoprotein complex, intracellular organelle, cytoplasm, organelle lumen, ribosomal subunit, etc., most of which showed a downward trend, accounting for 65.6%. The results of the “molecular function” GO enrichment showed that the higher frequency molecular functions are nucleoside binding, transferase activity, the transfer of phosphorus-containing groups, and hydrolase activity, and so on. GO enrichment analysis showed that in the BAT sample, there were 148 DEGs annotated in biological processes, of which approximately 56.8% of DEGs were up-regulated and 43.2% of DEGs were down-regulated. In cellular components, 177 DEGs (57 up, 120 down) were annotated and 25 DEGs (14 up, 11 down) were annotated in the molecular function (Figure 7B, Table S5).

In biological processes, DEGs were highly enriched in macromolecule metabolic processes, transports, organonitrogen compound metabolic processes, and biosynthetic processes. Similar to the BST group, the BAT sample has a higher frequency of cellular component subclasses, which are mainly involved in the intracellular, intracellular part, intracellular ribonucleoprotein complex and its membrane function. In the molecular functional classification, DEGs are enriched in nucleoside binding, transferase activity, the transfer of phosphorus-containing groups, and hydrolase activity. Transcriptome analysis was also occasionally applied to the study of biological control mechanisms [26,27]. *Rhizoctonia solani* was treated with the *Serratia proteamaculans* and *S. plymuthica*, resulting in a difference of 462 and 242 genes, respectively, and these gene enrichment assays and functional classification of DEGs were annotated by the GO term [26]. The up-regulated GO terms were the threonine-type endopeptidase activity, oxidation-reduction process, biosynthetic process, pyruvate metabolism, and proteolysis involved in the cellular protein catabolic process, while the under-regulated GO terms included the DNA-dependent transcription, purine ribonucleotide metabolic process, nucleoside-triphosphatase activity, ribosome biogenesis, and ribosome and DNA binding. *Saccharothrix yanglingensis* was used for the biological control of *Valsa mali*. The transcriptome revealed that most of the glycoside hydrolase genes were down-regulated, including three key causative genes and the pectinase gene. The GO term revealed that the DEGs were down-regulated and involved in the dcatalytic activity, transmembrane transport, and hydrolase activity [27]. A transcriptome analysis was carried out to mine the downstream genes, whose expression levels were affected by melatonin [28]. After treating them with melatonin, the carbohydrate metabolism, lipid metabolism and amino acid metabolism of *P. infestans* was altered in the transcriptome data. The down-regulation of the rate-limiting enzyme 6-phosphofructokinase and fatty acid synthase would reduce or suppress the carbohydrate and lipid metabolism. Mei et al. [29] applied the combination of transcriptomic and proteomic approaches to explore the novel antifungal mechanisms of Benzothiazole against *P. capsici*. A total of 1,071 DEGs were identified, and they were related to the biological regulation, growth, organelle part, and cell part, and the single-organism process was down-regulated.

GO analysis demonstrated that four stress response genes, two detoxification-related genes, two-cell membrane genes, and three cytoskeletal organization genes were significantly down-regulated. Two genes involved in the apoptosis of cell were significantly up-regulated by benzothiazole, and the results of transmission electron micrographs confirm the apoptosis of *P. capsici*. Phenazine-1-carboxylic acid (PCA)-producing *Pseudomonas* spp. have been found to be effective against numerous plants pathogenic organisms, including bacteria, fungi, and oomycetes, such as the causal agent of the bacterial blight of rice, and the oomycetes *P. infestans* [45,46]. Ye et al. [46] used transcriptomics to exploit the alteration in *P. infestans* in response to phenazine-1-carboxylic acid production by *P. fluorescens*, and the transcriptome analysis provided a better understanding of the molecular mechanisms, which might in turn contribute to developing and optimizing a biocontrol strategy against the late blight of potato. Transcriptomic analyses implied that the percentages of all *P. infestans*’ genes significantly changed due to *P. fluorescens,* and exogenically applied PCA increased as time progressed, from 50 to 61% and from to 32 to 46%. When applying an absolute cut-off value of ≧3-fold change, 207 genes were found to be remarkably differentially expressed. Gene ontology analysis revealed that the key functional genes of the DEGs are involved in major functions, like phosphorylation mechanisms, transmembrane transport, and oxidoreduction activities.

### 2.6. Validation of RNA-Seq Sequencing

**Figure 8:**
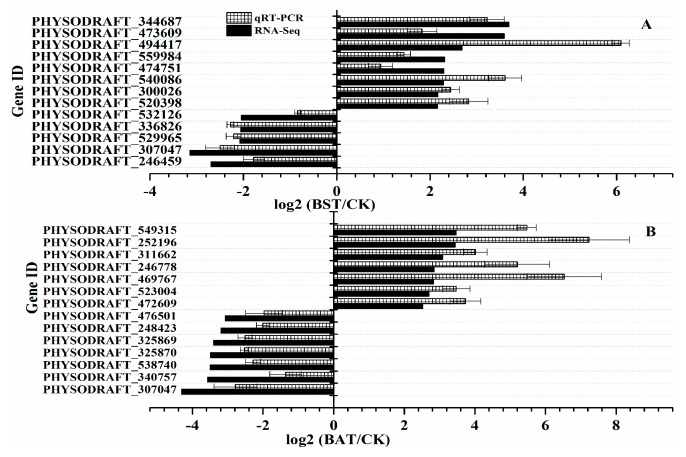
Comparison of gene expression patterns obtained using RNA-Seq and qRT-PCR validation. The *Y*-axis shows genes in three tissues validated in this study; *X*-axis shows the log2 ratio of expression in BST and BAT versus CK.

To verify the reliability of the RNA-Seq data, we randomly selected 14 genes in the BAT group and 13 genes in the BST group for quantitative RT-PCR (qRT-PCR) differential expression verification. Fold changes from qRT-PCR were compared with RNA-Seq expression analysis data, and the results are shown in Figure 8. The qRT-PCR results are in agreement with the RNA-Seq high-throughput sequencing data, indicating a similar expression pattern of up- and down-regulated genes, using RNA-Seq sequencing, and qRT-PCR tests.

### 2.7. Effect of B. amyloliquefaciens and B. subtilis on the Control of the Phytophthora Root Rot of Soybean

**Figure 9:**
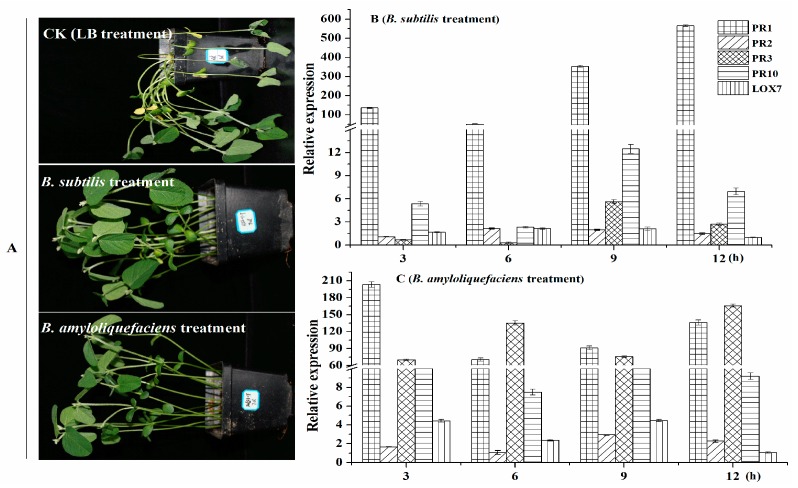
*B. amyloliquefaciens* and *B. subtilis* enhanced the resistance of soybean to *P. sojae.* (**A**) A soybean was given a 1 mL liquid culture of *Bacillus* (OD value = 0.8−1.0) five times. (**B** and **C**) The degrees of resistance genes expression in roots of 4-day old soybean seedlings were within 3−12 h after first root irrigation.

As shown in Figure 9, in the CK group, soybean seedlings have obvious lesions, and some of the soybean stalks and leaves were dry. However, the mildly susceptible potted soybean seedlings, under the hypocotyls showed a yellow-wild phenotype, and the base of the stem and the main root had mild brown lesions. However, for soybean seedlings treated with *B. subtilis* RSS-1 and *B. amyloliquefaciens* JDF3, only a small part of the hypocotyls showed yellowish or brownish black spots, and the rhizome had mild lesions. Their control effects of them were 65.5% and 70.7%, respectively. To further understand the effect of *Bacillus* on the disease resistance mechanism of soybean, we used the qPCR method to evaluate the expression of genes involved in soybean disease resistance (PR1, PR2, PR3, PR10, and LOX7) [47,48]. The sequence of quantitative primers is shown in Table S1. Compared with the control group, soybean treated with *B. subtilis* RSS-1 had significantly (*p* < 0.01) increased the expression levels of genes PR1, PR10, and LOX7, while the expression levels of genes PR2 and PR3 were slightly increased. After being treated with *B. amyloliquefaciens* JDF3, five disease resistance genes of the soybeans showed a significant up-regulation expression, except for PR2 (3 and 6 h) and LOX7 (12 h). Therefore, the two strains of *Bacillus* had an efficient control capability in relation to soybean phytophthora root, which was not only related to the inhibition of the growth of *P. sojae*, but also closely connected with the stimulation of soybean resistance [47,48], such as promoting the synthesis genes of phytoalexin and chitinase up-regulation [49].

### 2.8. B. amyloliquefaciens Fermentation Broth (BAFB) Activates Soybean Plant Defense Responses

**Figure 10:**
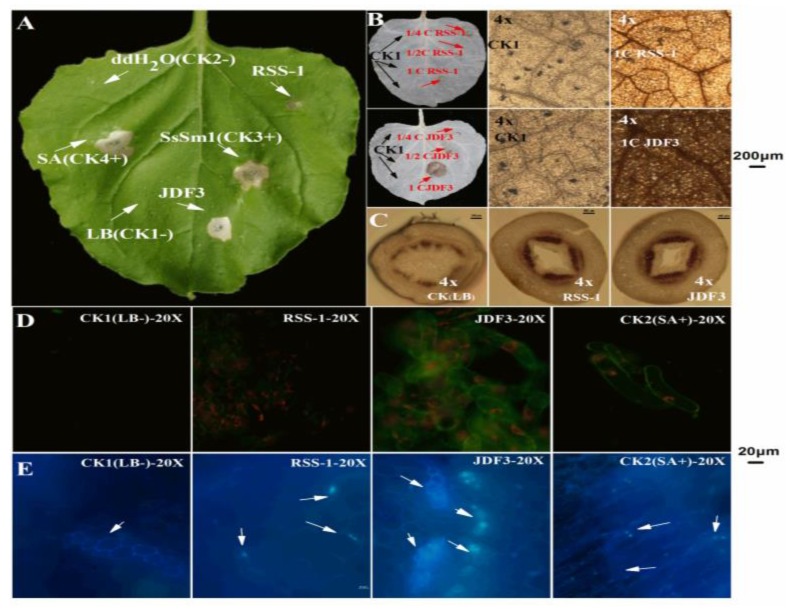
*B. amyloliquefaciens* fermentation broth (BAFB) activates soybean plant defense responses. RSS-1 and JDF3 were on behalf of fermentation broth of *B. amyloliquefaciens* JDF3 and *B. subtilis* RSS-1, which were degerming with millipore filter (0.22 μm). An LB liquid medium and dd H_2_O were separately injected as negative controls (−), and salicylic acid (SA) and SsSm1, an elicitor protein was injected as a positive control (+). B and C were taken under a four-fold microscope, while D and E were recorded with a 20-fold microscope. The green fluorescence was generated by the combination of the NO and detection probe, and the orange fluorescence was generated by chloroplast stimulated by a fluorescence microscope (Nikon, Tokyo, Japan, NIS-elements D, Nikon, Tokyo, Japan) light source (D).

As shown in Figure 10A, the negative control did not cause significant changes in the leaves, while the phenotype caused by *B. subtilis* RSS-1 fermentation broth was similar to that of the negative control. BAFB caused a hypersensitive reaction (HR) in tobacco leaves, which caused the HR phenotype to be consistent with the SA positive control (+). The above results indicate that BAFB might induce plant defense responses. In addition, previous studies have confirmed that protein elicitor (PeBA1) and lipopeptides secreted by *B. amyloliquefaciens* can induce plant defense responses [50,51]. Moreover, BAFB induced an active oxygen burst, and the DAB staining shown in Figure 10B. A negative control sample was injected on the left side of the tobacco leaves, which showed milky white after decolorization, while the injection site and veins showed a light-brown color under the microscope. Tobacco leaves treated with 4, 2, and undiluted *B. subtilis* RSS-1 fermentation broths (BSFB) were essentially no different, compared to the negative control treated with the LB liquid medium. The tobacco leaves treated with BAFB diluted two times and undiluted BAFB showed a brown color, which was significantly different from the white color. Microscopically, the tobacco leaves treated with BAFB were dark brown and the color was significantly darker than that of the control, indicating that the BAFB induced a large amount of active oxygen bursting.

After staining with CK soybean stalks, lignin binds to phloroglucin and appears dark red. Compared with the CK, the soybeans treated with two strains of *Bacillus* had a significantly deeper crimson and more numerous red complexes in the stem (Figure 10C). It can be seen from Figure 10C that *Bacillus* can promote the lignification of soybean, thereby further improving the control effect of soybean disease. NO is an important signaling molecule of soybean, and it plays an important role in response to elicitor induction to promote plant stress resistance [52]. An NO fluorescent probe kit was used to detect whether BSFB and BAFB could induce NO signal molecules in soybean single cells. No fluorescence signal was detected in the soybean single cells treated with the negative control, whereas the fluorescent signal in the soybean single cell sample treated with the positive control SA was strong (Figure 10D). The single-cell fluorescence signal of soybean treated with BSFB was slightly stronger than that of the negative control, which was significantly weaker than the positive control SA, indicating that it significantly failed to induce the production of soybean NO signaling molecules. However, the single-cell fluorescence signal of soybean treated with BAFB was significantly stronger than that of the negative control, and its effect was similar to that of the positive control SA. This phenomenon indicates that it can significantly stimulate the accumulation of NO in the soybean, and BAFB may affect the resistance of soybean. Additionally, BAFB induces callose deposition in soybean, and a yellowing nursery was observed (Figure 10E). The soybean sprouts treated with BAFB showed brighter blue particles under the microscope. Callose also plays an important role in the disease resistance process of plants such as soybean, and its deposition in plants can help plants respond to pathogen infection and abiotic stress (physical, chemical, environmental factors, etc.) [2,9,52].

## 3. Materials and Methods

### 3.1. Strains and Culture Conditions

Two bacteria with potential biocontrol effects, *B. amyloliquefaciens* JDF3 and *B. subtilis* RSS-1, were isolated, identified, and preserved by the mycology and plant fungal disease laboratory of college of plant protection, Anhui agricultural university. *P. sojae* P6497 was presented by Professor Wang Yuanchao of the Nanjing Agricultural University. The strain was cultured in a 10% (*v*/*v*) V8 solid medium for five days and used for the confrontation culture of *B. amyloliquefaciens* JDF3 and *B. subtilis* RSS-1. *P. sojae* discs were obtained with a 9 mm cork borer, then the two bacteria dishes were placed in the circles on the left and right sides of the dish diagram. The *B. amyloliquefaciens* JDF3 and *B. subtilis* RSS-1 single colonies, cultured in an LB solid medium at 37 °C for 24 h, were taken with an inoculation needle, and inoculation was performed by streaking 6 cm along the axis of the symmetry of the two circles. The culture dish was placed in a 25 °C incubator and cultured for five days. Each treatment was inoculated with 30 dishes, 10 dishes were grouped together, and finally, three biological replicates were obtained. The dishes of *P. sojae*, inoculated by the above method, without the inoculation of *Bacillus,* were used as the control. According to the published methods [2,17], inhibition rates (I) of mycelium growth was calculated as I = [(R1 − R2)/R1] × 100, where R1 is the control mycelium growth between two discs edge. R2 is the mycelium growth toward the *Bacillus*. The method for determining the EC50 value and the bacteriostatic rate of the fermentation broth refers to Cui et al. [53]. The fermentation broth of *B. amyloliquefaciens* JDF3 and *B. subtilis* RSS-1 was centrifuged at 10,000 rpm for 10 min, and the supernatant was filtered with 0.22 μm filter membrane. The ability of inhibiting germination of *P. sojae* cysts were evaluated according to the previous literature [44,54], and the plates, which contained 4% fermentation broth of *Bacillus*, were used. CK3 (V8) and CK4(V8 + 4%LB) were used as negative and positive controls.

### 3.2. RNA Extraction, cDNA Library Preparation. and Transcriptome Sequencing

Referring to the method reported by Gkarmiri et al. [26], under sterile conditions, a scalpel was used to scrape the *P. sojae* hygrofilament, with a width of 1.5 ± 0.2 cm, along the outermost axis of the dish, along the axis of symmetry. Into one group, 10 dishes were mixed. Liquid nitrogen was used to freeze them, and they were prepared for RNA extraction. RNA extraction was performed using the takara RNA extraction kit, following the method provided in the kit. The concentration and purity of the RNA samples were quantified using a spectrophotometer (NanoDrop ND-1000; Thermo Fisher Scientific, Waltham, MA, USA), and the RNA degradations of nine samples were assessed in 1% agarose gels. The RNA integrity was assessed using the RNA Nano 6000 Assay Kit of the Agilent Bioanalyzer 2100 system (Agilent Technologies, CA, USA). Sequencing libraries were generated using NEBNext^®^ Ultra™ RNA Library Prep Kit for Illumina^®^ (NEB, Ipswich, MA, USA), following the manufacturer’s recommendations. The effective concentration of the library was accurately quantified using QPCR to ensure the library quality, and then cDNA library sequencing was conducted with an Illumina high-throughput sequencing platform (HiSeqTM2500) by Guangzhou Saizhe Biotechnology Co., Ltd. (Guangzhou, China).

### 3.3. RNA-Seq Data Analysis

High-quality clean data can be obtained by the quality control of the raw data output from the sequencing platform, deleting the linker sequence and sequencing primer sequences, and filtering low-quality read lengths. The clean data were compared to the *P. sojae* 3rd edition reference genome by the software, TopHat2, and the mapped data were assembled using the software, Cufflinks. The assembly results of multiple samples were combined with Cuffmerge. The expression and transcripts of genes were quantified using the Cuffdiff module, and the expression level of the gene was calculated by locating the reads each 1 million maps to the reads per megascript of the transcript per Million fragments mapped (FPKM). The differential expression analysis and calculation of the genes were performed using the EBSeq software, and the false discovery rate (FDR) of each transcript was counted. The fold change (FC) of the gene expression was calculated from different samples, using FDR and log2FC to screen differential genes. The treatment group was considered to have a significant differential expression relative to the control, and the screening conditions were FDR < 0.05 and |log2FC| > 1.

### 3.4. Gene Annotation

The Gene Ontology (GO) annotation is based on the significant enrichment of GO functions to analyze DEGs and related gene modules for bioinformatics analysis. Referring to the annotation information in the non-redundant (Nr) database, the Blast2 GO software (version 3.0, https://www.blast2go.com/, BioBam Bioinformatics S.L., Valencia, Spain) was used to perform GO Annotation on the core genes in the differentially expressed genes and the co-expressed gene modules, and the WEGO software was used to annotate and statistical the GO function classification of all genes. It covers three aspects of biology: the cellular component, the molecular function, and the biological process. The Kyoto Encyclopedia of Genes and Genomes (KEGG) database (available online: https://www.kegg.jp/) can systematically classify and annotate the metabolic pathways of genes and classify and study genes and their expression information at a general level. The KO-BAS software (version 2.0, KOBAS, Surrey, UK) was used to detect the enrichment of DEGs in the KEGG pathway, and the biological function of the specific gene of *P. sojae* was considered and evaluated at a macro level.

### 3.5. Quantitative Real-Time PCR (qRT-PCR)

The remaining *P. sojae* and soybean samples were taken for RNA extraction and purification treatment experiments and then reverse transcribed into cDNA for use. Twenty-seven DEGs were randomly selected and verified by relative quantitative expression using fluorescence quantitative PCR. To verify their resistance changes after stimulation by *B. subtilis* RSS-1 and *B. amyloliquefaciens* JDF3, the genes (PR1, PR2, PR3, PR10, and LOX7) of soybean [53,54] were used for qRT-PCR. The ratio of the degree of gene expression at different time periods to the degree of gene expression of the untreated sample was used to evaluate the soybean resistance; that is, the relative expression level of the resistance gene. The genes, *Physo1_1* 108986 (PsActinA for *P. sojae*) [51,52] and *EFla* (Actin for soybean) [53,54], were used as actin for qRT-PCR. Moreover, all the qRT-PCR primers were designed with the software Premier 6.0, and synthesized by Nanjing Qingke Biological Co., Ltd. (Nanjing, China). The primer sequences used are shown in Table S5, and the qRT-PCR verification test for the differential gene expression were carried out according to the method described in the takara kit. The qRT-PCR system (25 μL) is as follows: 2 μL of template cDNA, 12.5 μL of SYBR green mixture, 8.5 μL of dd H_2_O, and 1 μL of each of the upstream and downstream primers. The reaction procedure was as follows: 95 °C, 30 s; 39 cycles (95 °C for 5 s, 60 °C for 30 s). The above reaction procedure was carried out using ABIPRISM 7300 (Applied Biosystems, Foster, CA, USA), and the obtained product was obtained after the PCR amplification reaction was completed. To obtain the dissolution curve, the temperature was increased from 65 °C to 95 °C.

### 3.6. Effect of B. amyloliquefaciens and B. subtilis on the Control of Phytophthora Root Rot of Soybean

Soybean greenhouse cultivation referred to the method reported by Lu et al. [2], with slight modifications. Four days after sowing, the soybean seedlings were given a one milliliter liquid culture of *B. subtilis* RSS-1 or *B. amyloliquefaciens* JDF3 (OD value = 0.8–1.0) to a soybean every two days, and roots were implemented a total of five times. The effects of *B. amyloliquefaciens* and *B. subtilis* on the control of the Phytophthora root rot of soybean were evaluated with the method described by Lu et al. [2] and Pawlowski et al. [49]. The control effects (CE) was calculated as CE = [(disease incidence _control_-disease incidence _treatment_)/disease incidence _control_] × 100%, and disease incidence was based on whether the disease/pathogen was not observed (rating = 0) or observed (rating =1) and converted to percent incidence. Severity ratings were recorded from 0 to 5 using a pre-transformed scale (0 = no visible detection, 1 = 1–10%, 2 = 11–35%, 3 = 35–65%, 4 = 66–90%, and 5 = 91–100% of the stem or sampled area covered).

### 3.7. BAFB Activates Soybean Defense Responses

The fermentation broth was centrifuged at 10,000 rpm for 10 min, and the supernatant was filtered with a 0.22 μm filter membrane. To verify whether *B. subtilis* RSS-1 and *B. amyloliquefaciens* JDF3 could induce plant resistance, we injected *Bacillus* fermentation broth into *Nicotiana benthamiana* leaves. An LB liquid medium and dd H_2_O were separately injected as negative controls (−), and salicylic acid (SA) [55] and SsSm1 [56], an elicitor protein, was injected as a positive control (+). The evaluation of the active oxygen burst was carried out with DAB staining [50]. The lignin was detected with a phloroglucinol kit based on instruction from Qingdao JSK Biotechnology Co., Ltd. (Qingdao, Shangdong, China). An NO fluorescent probe kit (Biyuntian Biotechnology Co., Ltd.,Shanghai, China) was used to detect the NO molecules in soybean single cells. Moreover, the callose staining referred to the method reported by Lu et al. [2].

## 4. Conclusions

*B. amyloliquefaciens* JDF3 and *B. subtilis* RSS-1 could inhibit mycelial growth, the germination of the cysts, and the swimming of the motile zoospores. Comparative transcriptomics was applied to elucidate the molecular mechanism of the biological control. A total of 1616 DEGs were detected. Most DEGs participated in inhibiting the activity of ribosome. A pot experiment indicated that *B. amyloliquefaciens* and *B. subtilis* enhanced the resistance of soybean to *P. sojae*, and their control effects were 70.7% and 65.5%, respectively. In addition, *B. amyloliquefaciens* fermentation broth could induce an active oxygen burst, NO production, callose deposition, and lignification.

## Figures and Tables

**Table 1:** Summary of the quality of transcriptomic sequencing data of *P. sojae.*

Samples	Raw Reads	Clean Reads	Q30 (%)	Total Reads	Mapped Reads	GC (%)
CK1	44778932	43841202	86.19	39868976	24694246	56.56
CK2	47469548	45251026	85.26	44950158	25585591	53.88
CK3	72226262	68948564	85.97	68250198	34299648	54.2
BST1	47339402	46338742	84.74	46161358	24371232	56.87
BST2	53546758	52564124	83.62	52223646	12675947	48.25
BST3	44294010	43117708	84.12	42928882	14629394	48.64
BAT1	51208004	50159286	85.89	49893596	29622070	57.73
BAT2	51667698	50872290	85.20	50318686	10625344	55.25
BAT3	46427178	45170256	85.48	44662734	15649105	56.96

CK1 = control 1, CK2 = control 2, and CK3 = control 3; BST1, BST2, and BST3 are three biological repeats of BST group. BST group = *P. sojae* samples treated with *B. subtilis* RSS-1. BAT1, BAT2, and BAT3 are three biological repeats of BAT group. BAT group = *P. sojae* samples treated with *B. amyloliquefaciens* JDF3. *P. sojae* was challenged with *B. amyloliquefaciens* and *B. subtilis* at 25 °C for five days.

## Supplementary Materials

Supplementary materials can be found at https://www.mdpi.com/1422-0067/20/12/2908/s1.
